# Effect of Plasma-Activated Water (PAW) on the Postharvest Quality of Shepherd’s Purse (*Capsella bursa-pastoris*)

**DOI:** 10.3390/foods13050703

**Published:** 2024-02-26

**Authors:** Lingming Xiong, Lei Feng, Meimei Nie, Dajing Li, Zhongyuan Zhang, Chunquan Liu, Zhuqing Dai, Yadong Xiao, Yayuan Xu

**Affiliations:** Institute of Agro-Product Processing, Jiangsu Academy of Agricultural Sciences, Nanjing 210014, China; xionglingming219@163.com (L.X.); fljaas@126.com (L.F.); nmm1011@163.com (M.N.); zzyszy2012@163.com (Z.Z.); liuchunquan2009@163.com (C.L.); bamboodzq@163.com (Z.D.); xyd15838746910@163.com (Y.X.); xuyayuanxyy2008@126.com (Y.X.)

**Keywords:** shepherd’s purse, plasma-activated water, postharvest quality, microbial growth, aroma characteristics

## Abstract

Plasma-activated water (PAW) treatment is an effective technique for the quality retention of fresh vegetables with cold atmospheric plasma using controllable parameters. This study investigated the effect of PAW on the postharvest quality of shepherd’s purse (*Capsella bursa-pastoris*). The results displayed that PAW treatment with an activation time of 5, 10, 15, and 20 min reduced the yellowing rate and weight loss of the shepherd’s purse during 9 days of storage. Compared with untreated samples, PAW treatment at different times reduced the number of total bacteria, coliform, yeast, and mold by 0.18–0.94, 0.59–0.97, 0.90–1.18, and 1.03–1.17 Log CFU/g after 9 days of storage, respectively. Additionally, the treatments with PAW-5 and PAW-10 better preserved ascorbic acid, chlorophyll, total phenol, and total flavonoid contents. They also maintained the higher antioxidant and CAT activity and inhibited the formation of terpenes, alcohols, and nitrogen oxide compounds of the shepherd’s purse at the end of storage. The microstructural result illustrated that the cells of the shepherd’s purse treated with PAW-5 and PAW-10 were relatively intact, with a small intercellular space after storage. This study demonstrated that PAW treatment effectively improved the postharvest quality of shepherd’s purse.

## 1. Introduction

Shepherd’s purse (*Capsella bursa-pastoris*) is a member of the family of Brassicaceae and is widely spread throughout the world [1]. It is a good source of flavonoids, sulforaphane, amino acids, polypeptides, vitamins, and organic acids, which exhibit good antioxidant, antibacterial, antitumor, and anti-inflammatory activity [2,3]. With the increasing demand of consumers for a healthy diet, shepherd’s purse, with high nutritional values and a special flavor, is a top-notch edible and medicinal wild herb that has received extensive attention. However, shepherd’s purse exhibits a high water content, strong seasonality, and a relatively short shelf life. Its appearance, flavors, and nutrients are rapidly lost after harvesting because of microbiological decay, water loss, and physiological deterioration [4]. Previous studies showed that shepherd’s purse has a shelf life of less than 2 days at 20 °C and 4 days at 5–10 °C [5,6]. Thus, postharvest preservation technology plays an important role in maintaining the postharvest qualities and extending the shelf life of shepherd’s purse.

Plasma-activated water (PAW) is produced by using cold plasma to treat distilled water for a period of time, which alters the physicochemical properties of water, such as the oxidation-reduction potential (ORP), electrical conductivity, and pH [7]. It contains a high number of nitrogen and reactive oxygen species (RONS), which derive from the combined action of low pH and have been proven to be effective in microbial inactivation [8]. PAW treatment reduces the surface browning of fresh-cut apples without affecting their hardness and titratable acidity. In addition, there was no significant change in antioxidant content and free radical scavenging activity between the PAW treatment group and the control group. Compared to the traditional heat and 1-methylcyclopene treatments, PAW treatment is a non-thermal, relatively milder, and environment-friendly food preservation method. It has been successfully employed to improve the shelf life of heat-sensitive vegetables and fruits [9,10]. Previous research suggested that PAW treatment could be an effective technique for the quality retention of fresh vegetables, such as the inhibition of yellowing, wilting, and microbial growth, resulting in better retention of bioactive compounds [11,12]. Zhao, Wang, & Ma [13] also showed that the physicochemical properties of the water could be changed with the increased plasma activation time, which influenced the quality of the product treated with PAW.

However, to the best of our knowledge, there is limited research on the application of PAW treatments for improving the postharvest quality of shepherd’s purse. This study intends to investigate the effects of PAW treatments on the postharvest quality of shepherd’s purse and evaluate aroma characteristics and microstructural features during storage. This will improve the technical support for the postharvest fresh-keeping treatment of shepherd’s purse.

## 2. Materials and Methods

### 2.1. Generation of PAW and Measurement of Physicochemical Properties

**Figure 1:**
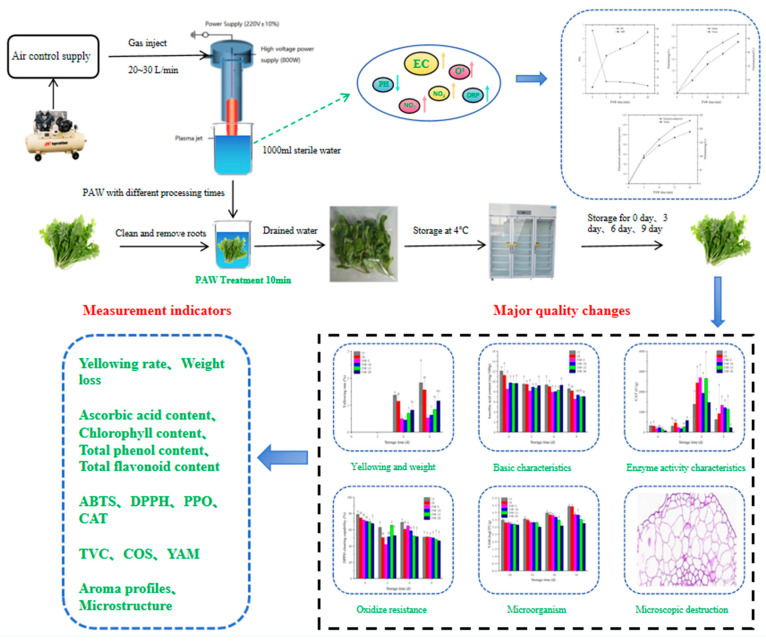
A schematic diagram of the experimental device and plasma equipment for treating shepherd’s purse.

As shown in Figure 1, the PAW generation consisted of a high-voltage generator (PG-1000Z/D, Nanjing Sunman Electronics Co., Ltd., Nanjing, China.), a plasma jet (800 W, 20 kHz), air control supply, and quantitative sterilization water. Compressed air is generated by an air compressor, producing an air pressure of 0.18 Mpa for working gas. The entire gas flow rate is controlled between 20 and 30 L/min. The head of the plasma jet contains a conical head, which is used to fully react with the generated plasma and water. The conical head was placed 5 mm under the water level during use, and every 1000 mL of sterile distilled water was activated by cold plasma for 5, 10, 15, and 20 min. Activated water was used after cooling to room temperature.

The oxidation-reduction, pH, and electrical conductivity (ORP) of PAW were determined using a multimeter (DZS-708T, Shanghai, China). The concentrations of nitrate (NO^−3^) and nitrite (NO^−2^) of PAW were measured via the spectrophotometry method [14]. The O_3_ was measured by a bench ozone meter (DOZ30, Guangzhou, China).

### 2.2. Preparation of Shepherd’s Purse and Treatment

#### 2.2.1. Preparation of Shepherd’s Purse

Fresh shepherd’s purse was harvested in a greenhouse in Nanjing, China. All the vegetables were taken from a different plot in the morning. Harvested shepherd’s purse was packed in plastic bags, and then it took 0.5 h for vegetable collection and 1 h for transportation to the laboratory at the Jiangsu Academy of Agricultural Sciences. Shepherd’s purse in a uniform size and without any observed defects was selected, washed, and then used for PAW treatment.

#### 2.2.2. Procedures for Treating Shepherd’s Purse

A total of 2340 g of shepherd’s purse was selected without any observed defects. Immediately after harvest (day 0), shepherd’s purse in each treatment group were air-dried in a biosafety cabinet for 30 min after washing and immersing. In total, 1080 g of shepherd’s purse was used and separately packaged for the following treatment in triplicate: (1) microbe group (total 360 g) and (2) weighing group (total 720 g). The remaining 1260 g of shepherd’s purse was separated into three groups as follows for treatment in triplicate: (1) untreated group (total 210 g), (2) control group (total 210 g), and (3) treated group (total 840 g). All samples were stored at 4 ± 1 °C and 90% ± 5% relative humidity for 9 days and analyzed every 3 days.

The shepherd’s purse was randomly assigned to untreated, control, and treated groups. For the PAW treatment, the shepherd’s purse (100 g in each group) was immersed in 1 L of PAW at different activation times (5, 10, 15, and 20 min) for 10 min, named PAW-5, PAW-10, PAW-15, and PAW-20, respectively. The shepherd’s purse without any treatment and immersed in 1 L of distilled water for 10 min were defined as UT and CK, respectively.

### 2.3. Weight Loss and Yellowing Rate

The mass of the shepherd’s purse was weighed every 3 days, and the weight loss of samples was expressed as the percentage of the reduced mass and initial mass (%). The weight loss of the shepherd’s purse was recorded according to the method of Chen et al. [15].

The yellowing rate of the shepherd’s purse was calculated every 3 days, and the yellowing rate of samples was expressed as the percentage of number of yellowing leaves and the total leaf (%). The yellowing rate of the shepherd’s purse was recorded according to the method of Cai et al. [16] with a slight modification. The yellowing rate was calculated by the following equation:

(1) $$Y=\Sigma (Y_{m}\cdot N_{m})/(Y_{h}\cdot N_{a})$$

where *Y* was the Yellowing Index, *Y_m_* and *Y_n_* represented the yellowing level value and the total number of flower heads, and *N_m_* and *N_a_* represented the number of flower heads corresponding to the yellowing level value and the highest level value of yellowing.

There were five levels of yellowing value: Level 0 (the surface of the flower ball had no yellowing, and the flower buds were firm); Level 1 (partial yellowing and the yellowing area accounted for 25% of the flower ball area); Level 2 (the flower buds turned yellow and the yellowing area accounted for 26% to 50% of the flower ball area); Level 3 (the flower buds turned yellow and the yellowing area accounted for 51% to 75% of the flower ball area); Level 4 (the flower buds turned yellow severely and the yellowing area accounted for 76% to 90% of the flower ball area); Level 5 (the flower buds almost completely lost their green color and the yellowing area accounted for more than 90% of the flower bud area).

### 2.4. Nutrient Content

The ascorbic acid content was examined following the method of Kim et al. [17]. The ascorbic acid content was expressed as mg/100 g FW. A total of 2.5 g of sample was homogenized within 25 mL of 0.25% metaphosphate with pre-cooling at 4 °C in advance, followed by centrifugation at 9000 r/min for 5 min, and filtered through a 0.45 µm filter.

The chromatographic separation was achieved using a ZORBAX 300SB-C18 (250 mm × 4.6 mm × 5 µm) column and set at 25 °C with a mobile phase of 0.03 mol/L of phosphoric acid (0.8 mL/min). Data were calculated according to a pre-established standard curve, and the result was expressed as mg/100 g FW.

The chlorophyll content was examined according to the method of Jia et al. [18]. A total of 1 g of shepherd’s purse was added to 95 mL/100 mL of ethanol, and a small amount of quartz sand and calcium carbonate was grounded in a mortar. Then, the ground mixture was filtered, the residue was grounded, and the filtration was repeated until the solution turned colorless and simmered for 3–5 min. The filtered material was collected into a 25 mL volumetric flask and filled with 95% ethanol to a certain volume.

The chlorophyll content of the shepherd’s purse was examined by a UV spectrophotometer at 665 nm and 649 nm. The total chlorophyll content was calculated as the sum of the chlorophyll a and chlorophyll b content; the chlorophyll a, b, and total chlorophyll were calculated as follows:

Ca = 13.95 × D_665_ − 6.88 × D_649_, (2)

Cb = 24.96 × D_649_ − 7.32 × D_665_, (3)

CT = Ca + Cb,(4)

where Ca, Cb, and CT are contents of chlorophyll a, b, and total chlorophyll, respectively. D_649_ and D_665_ were the absorbance values at 649 and 665 nm, respectively. The results were expressed as g/kg FW.

The total phenolic content (TPC) of the shepherd’s purse was determined with a Folin–Ciocalteau reagent by a colorimetry assay [19] with slight alterations. A total of 1 mL of sample extracts and 5 mL of distilled water were mixed. Then, 1 mL of Folin reagent, diluted by 1 time, was mixed. A total of 3 mL of 7.5% Na_2_CO_3_ was added after 5 min. Finally, all solutions were mixed well and incubated for 2 h in a dark environment. With 50% ethanol taken as a blank, the absorbance was calculated at 765 nm. The TPC was expressed as mg (gallic acid equivalent)/100 g FW.

The total flavonoid content (TFC) of the shepherd’s purse was determined by a colorimetry assay according to the method of Feng et al. [20]. Firstly, 3 mL of sample extracts and 0.5 mL of 5% NaNO_2_ were mixed well after 6 min. Then, 0.5 mL of 10% Al(NO_3_)_3_ was mixed after 6 min. Finally, 4 mL of 4% NaOH was added, and the volume was adjusted to 10 mL with distilled water, thoroughly mixed, and incubated at 25 °C for 10 min. The absorbance was calculated at 509 nm, with 50% ethanol as the blank. The TFC was expressed as mg (rutin equivalent)/100 g FW.

### 2.5. Antioxidant Properties

The DPPH and ABTS radical scavenging rates were used to assess the antioxidant properties of the shepherd’s purse. The DPPH and ABTS were examined following the method of Muley & Singhal [21] with a slight modification. The samples (1 g) were placed in deionized water (20 mL), ground into pulp in an ice bath, and centrifuged for 15 min at 4 °C and 12,000× *g*; the supernatant was collected.

In brief, for a configuration of 0.2 mmol/L DPPH, the volume was adjusted to 100 mL with absolute alcohol. A total of 100 µL of the supernatant and 100 µL of the 0.2 mmol/L DPPH were mixed well and incubated for 30 min in a dark environment. The absorbance was read at 517 nm against the reagent blank. The DPPH radical scavenging activities were calculated by the following equation:

I% = [(A_control_ − A_sample_)/A_control_] × 100%, (5)

where A_control_ and A_sample_ refer to the absorbance of control and sample extract, respectively.

A total of 20 µL of the supernatant and 180 µL of ABTS radical working solution were incubated for 60 min in a dark environment. The absorbance was read at 734 nm against the reagent blank. The ABTS radical scavenging activities were calculated by the following equation:

I% = [(A_o_ − A_i_)/A_o_] × 100%, (6)

where A_o_ and A_i_ refer to the absorbance of the control and sample extract, respectively.

### 2.6. Enzymatic Activities

The polyphenoloxidase (POD) and catalase (CAT) activity of the shepherd’s purse were determined according to the method of Lin et al. [22] with a slight modification.

The POD activity was determined spectrophotometrically using guaiacol. A total of 2 g of shepherd’s purse was added to liquid nitrogen and ground in a mortar with a small amount of quartz sand and 8 mL (pH = 6.8) of acid-nitrate after 20 min at 4 °C. It was centrifuged for 15 min at 4 °C and 12,000× *g*, and the supernatant was collected.

A total of 2.1 mL of phosphate buffer (pH 6.8) and 0.6 mL of Catechol (0.1 M) was mixed. The mixture was incubated at 30 °C for 5 min, and 0.3 mL of supernatant was then added to interrupt the enzymatic reaction. The POD activity was determined using a UV–Vis spectrophotometer at 420 nm. The POD activity was expressed as U/g FW.

In this paper, the kit method (BC0205-100T/96S, Solarbio) was chosen as the method to determine the CAT activity; CAT activities were expressed as U/g FW.

### 2.7. Microbial Growth

The microbial growth, including the total viable count (TVC), coliforms (COS), and yeast and mold (YAM) of the shepherd’s purse, were determined by standard enumeration methods on a microbial media (PDA, Scharlau Chemie, SA., Barcelona, Spain).

The shepherd’s purse samples (5 ± 0.1 g) from the microbe group were homogenized with Peptone Physiological Solution (PPS) (1 g/L peptone (Sinopharm Group Co., Ltd., Shanghai, China) + 8.5 g/L NaCl (Sinopharm)) in a sterile stomacher bag. Then, the decimal dilution series was prepared in PPS, and the appropriate dilutions were plated on different media for the enumeration of different microbial populations. The whole process was performed in a laminar flow bench (SJ-CJ-1D, Suzhou Purification Equipment Co. Ltd., Suzhou, China). The total viable count (TVC, Hopebio, Qingdao, China) was determined by spread plating on plate count agar (PCA, Hopebio) plates and incubated at 30 °C for 3 days. The cell counts of coliforms were determined on a MacConkey agar (Oxoid, Hampshire, UK) and incubated at 37 °C for 3 days. Yeasts and molds (Y&M, Hopebio) were counted on a Rose Bengal Chloramphenicol Agar (RBCA; Hopebio) and incubated at 22 °C for 5 days. All microbial counts were expressed as log CFU/g.

### 2.8. Aroma Characteristics

The aroma characteristics of the shepherd’s purse were examined by e-nose according to the method of Feng, Zhang, Bhandari, & Guo [23]. There were 10 metal oxide semi-conductor sensors in the sensor array system (Airsense Analytics Co., Ltd., Schwerin, Germany. Table 1). The shepherd’s purse (6 g) was grated by a ball mill (50 Hz, 60 s, JX-2G, Jingxin, Shanghai, China), then the shepherd’s purse (2 g) was placed in an airtight bottle (50 mL) and equilibrated for 30 min at room temperature to determine the flavor. The airflow, testing time, and cleaning time were 1 L/min, 120 s, and 120 s, respectively.

### 2.9. Microstructure

The periodic acid–Schiff (PAS) reaction method was used to determine the microstructure of the shepherd’s purse. The sample (1 cm × 1 cm) was placed into a formaldehyde-acetic acid-ethanol fixative. The paraffin section was made and sliced with a thickness of 3 μm. The slices were washed in the selected buffer and dehydrated through an ethanol series. Then, the slices were stained with a Schiff reagent for 30 min, washed with distilled water for 5 min, and sealed with a neutral gum. The microstructure observations of the shepherd’s purse were carried out on a microscope. The magnification was set at 40×, and three slides were observed for each treatment group.

### 2.10. Statistical Analysis

All the experiments were performed in triplicates. A One-way ANOVA was used with SPSS (Version 13.0 Inc., Chicago, IL, USA) to analyze the data, and the statistical difference was obtained with a Duncan’s test at *p* < 0.05.

## 3. Results

### 3.1. Physicochemical Properties of PAW

**Figure 2:**
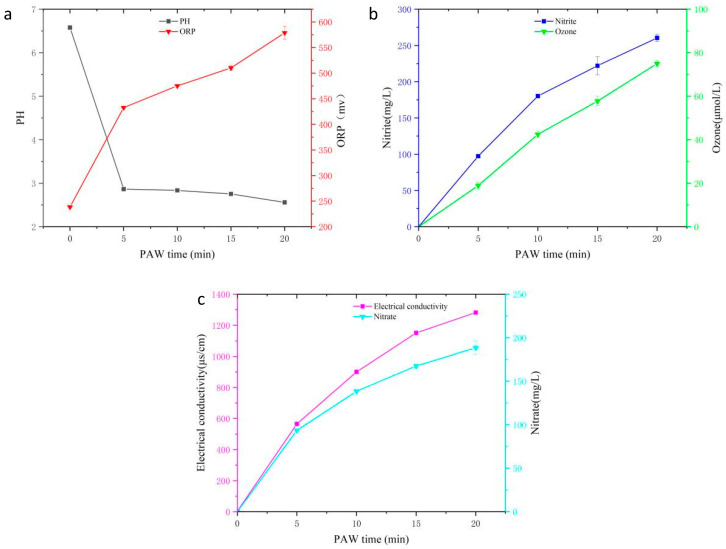
Characterization of PAW after different cold plasma activation times: (**a**) pH and ORP, (**b**) nitrite and ozone, and (**c**) electrical conductivity and nitrate.

The basic characterization of PAW with different cold plasma activation times is shown in Figure 2. Generally, the values of all the measured parameters (pH, ORP, O_3_, EC, NO^−2^, and NO^−3^) depended on the plasma activation time. The pH values of the PAW are shown in Figure 2a, which achieved approximately 2.8 units and dropped quickly during the first 5 min of plasma activation. As the processing time increased, the pH values decreased slowly. The results were in accordance with previous studies [24,25]. ORP is considered an important factor affecting microbial inactivation and indirectly indicates the level of the overall reactive oxygen species (ROS) in the PAW. The ORP values of PAW were raised from 232.30 to 576.16 mv as the plasma activation time increased from 0 to 20 min (Figure 2a). In comparison, the ORP values showed an upward trend as the processing time increased. The results of this study indicated that the PAW generated abundant active ions, including ROS and other active substances, which contributed to its antibacterial activity.

In order to identify the species of antibacterial activity in the PAW, quantitative and qualitative analyses were conducted on NO^−2^ and NO^−3^. As shown in Figure 2b,c, the concentrations of NO^−2^ and NO^−3^ showed upward trends as the processing time increased. With a processing time of 20 min, the concentrations of NO^−2^ and NO^−3^ reached maximum values of 190.12 mg/L and 248.53 mg/L, respectively. The concentration of NO^−3^ was much higher than that of NO^−2^ at the same processing time. This result was similar to the research of Choi et al. [10]. While NO^−2^ and NO^−3^ are major species of reactive nitrogen species (RNS), this active substance may cause oxidative damage to microorganisms through DNA destruction and the inhibition of lipid catalase and enzyme activity [10].

Furthermore, electrical conductivity (EC) can reflect the changes in the active ion content of water after plasma treatment, changing the conductivity of water, which is related to active oxygen and nitrogen [26]. The EC value increased with the increase in reaction time and reached a maximum value of 1302.25 μS/cm at the processing time of 20 min (Figure 2c). During the discharge process, a certain amount of ozone was generated (Figure 2b). As the treatment time increased, the ozone content almost linearly increased and reached a maximum value of 73.36 μmol/L at the processing time of 20 min.

### 3.2. Effect of PAW Treatment on the Yellowing Rate and Weight Loss of Shepherd’s Purse

**Figure 3:**
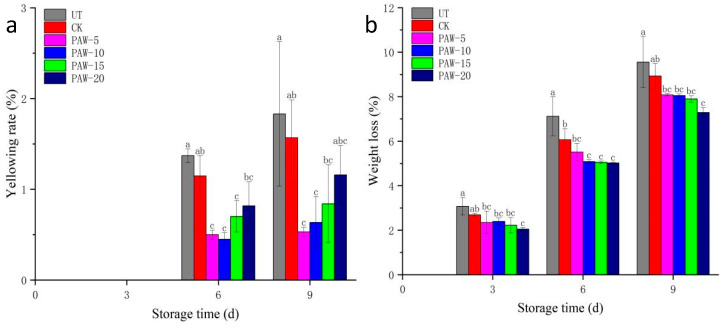
Effect of PAW on the yellowing rate and weight loss of shepherd’s purse during storage: (**a**) yellowing rate and (**b**) weight loss. a–c represents significant differences among different treatments at the same storage time (*p <* 0.05). UT (control); CK (control treatment immersed in 1 L of distilled water for 10 min); PAW-5 (control treatment immersed in 1 L of 5 min activated water for 10 min); PAW-10 (control treatment immersed in 1 L of 10 min activated water for 10 min); PAW-15 (control treatment immersed in 1 L of 15 min activated water for 10 min); PAW-20 (control treatment immersed in 1 L of 20 min activated water for 10 min); replicates (*n* = 3).

The yellowing rate and weight loss of the shepherd’s purse during storage are shown in Figure 3. The yellowing rate is a relatively important indicator for vegetables, which directly reflects the freshness of the shepherd’s purse and thus affects its commercial value [27]. As the storage time increased, its yellowing rate showed an upward trend. The PAW treatment group significantly reduced the yellowing rate of the shepherd’s purse. After 6 days of storage, the yellowing rates of the 5, 10, and 15 treatment groups were significantly lower than those of the CK treatment group (*p* < 0.05). The effect of the short-time treatment group was significantly better than that of the long-time group; the same trend was observed during the 9 day storage group. Compared to other treatment groups, the yellowing rate of the PAW-20 sample reached 1.59%. This indicated that PAW treatment with a long activation time actually increased its yellowing rate compared to other treatment groups.

Furthermore, the material consumption under water evaporation plays an important role in the weight loss of the shepherd’s purse, and the weight loss directly determines the quality of the shepherd’s purse during storage. As shown in Figure 3b, PAW treatment significantly reduced the weight loss, but unlike the effect of the yellowing rate, the higher the concentration, the better the reduction effect of weight loss was. Only the PAW-20 group showed significant differences compared to the CK group during storage for 3–9 days (*p* < 0.05). The weight loss of the UT and CK samples reached 9.55% and 8.93% after 9 days of storage, respectively. The weight loss of the PAW-20 sample was the lowest at 7.29% compared to other samples during storage. Previous research demonstrated that the weight loss of samples after PAW treatment decreased because the PAW effectively inhibited the respiratory rate.

### 3.3. Effect of PAW Treatment on the Nutritional Compounds of Shepherd’s Purse

**Figure 4:**
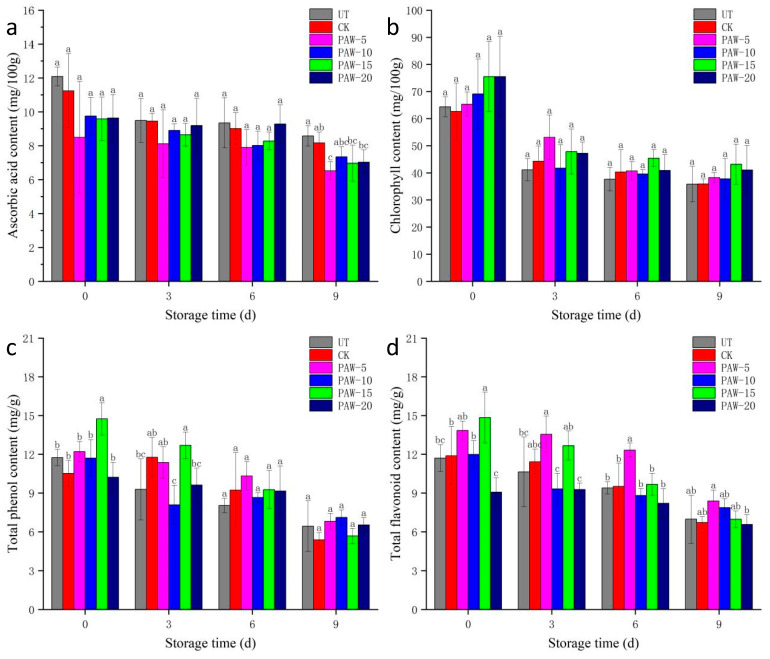
Effect of PAW on the nutritional compounds content of shepherd’s purse during storage: (**a**) ascorbic acid, (**b**) chlorophyll, (**c**) total phenol, and (**d**) total flavonoid. a–c represents significant differences among different treatments at the same storage time (*p <* 0.05). UT (control); CK (control treatment immersed in 1 L of distilled water for 10 min); PAW-5 (control treatment immersed in 1 L of 5 min activated water for 10 min); PAW-10 (control treatment immersed in 1 L of 10 min activated water for 10 min); PAW-15 (control treatment immersed in 1 L of 15 min activated water for 10 min); PAW-20 (control treatment immersed in 1 L of 20 min activated water for 10 min); replicates (*n* = 3).

The nutritional compounds, including ascorbic acid, chlorophyll, total phenol, and total flavonoid contents, of the shepherd’s purse during storage are shown in Figure 4. As shown in Figure 4a, the ascorbic acid contents of all samples continuously decreased with the extension of storage time. The result was in accordance with a previous study [28]. Furthermore, the loss rates of ascorbic acid in the UT and CK samples were 29.04% and 27.32%, respectively. The PAW-5 and PAW-10 samples had lower loss rates of ascorbic acid at 23.22% and 24.67%, respectively. In our study, the loss rates of ascorbic acid in the PAW-15 and PAW-20 samples were 27.30% and 27.06%, respectively, which were also lower than that of the UT and CK samples, but the difference was not marked. The results of the current study indicated that treatment by PAW-5 and PAW-10 effectively inhibited the loss of ascorbic acid during storage. Nevertheless, Misra & Jo. [29] suggested that the ozone and other active substances produced by CK treatment reacted chemically with ascorbic acid, resulting in its excessive consumption.

As shown in Figure 4b, the synthesis of chlorophyll in shepherd’s purse stopped after harvest, and its content continuously decreased with the prolongation of storage time. The initial content of the UT sample was 64.34 mg/100 g. After 9 days of storage, the chlorophyll content was 35.85 mg/100 g, with a high loss rate of 44.28%. Additionally, the treatment with PAW-20 had an adverse effect on the chlorophyll content of the shepherd’s purse. The chlorophyll content was only 54% of the initial value, and the loss rate reached 45.60% after 9 days of storage. The treatment with PAW-5 had a relatively good preservation of chlorophyll content, with a loss rate of only 41.50% (Figure 3b). Machala, Tarabova, Sersenova, Janda, & Hensel. [30] suggested that PAW contained ozone, and a certain amount of ozone inactivated the chlorophyll hydrolases and slowed down chlorophyll degradation. On the basis of the above results, it suggested that PAW treatment of a low fluence was conducive to delaying chlorophyll degradation to some extent by changing the chlorophyll metabolism pathway.

The TPC and TFC of shepherd’s purse with different treatments are shown in Figure 4c,d. At the 3 days of storage, it showed a trend of first increasing and then decreasing, but the fluctuation was relatively small; the results in this study were in agreement with reports by Yuanyuan [31]. The loss rates of the TPC and TFC for the shepherd’s purse treated with PAW were lower than that of the UT sample. The loss rates of TPC and TFC for the PAW-20 sample were 36.08% and 27.66% at 9 days of storage, respectively, which were higher than that of the CK sample. Compared with the CK sample, PAW treatment obviously increased the loss rates of total phenol and flavonoid content in the shepherd’s purse. Grzegorzewski, Ehlbeck, Schlueter, Kroh, & Rohn [32] suggested that the OH, O, and other parameters in PAW might cause the erosion of the epidermal layer of lettuce where flavonoid and other accumulated compounds in the central vacuoles of guard cells and epidermal cells were released and degraded.

### 3.4. Effect of PAW Treatment on the Antioxidant Activity and Enzyme Activity of Shepherd’s Purse

**Figure 5:**
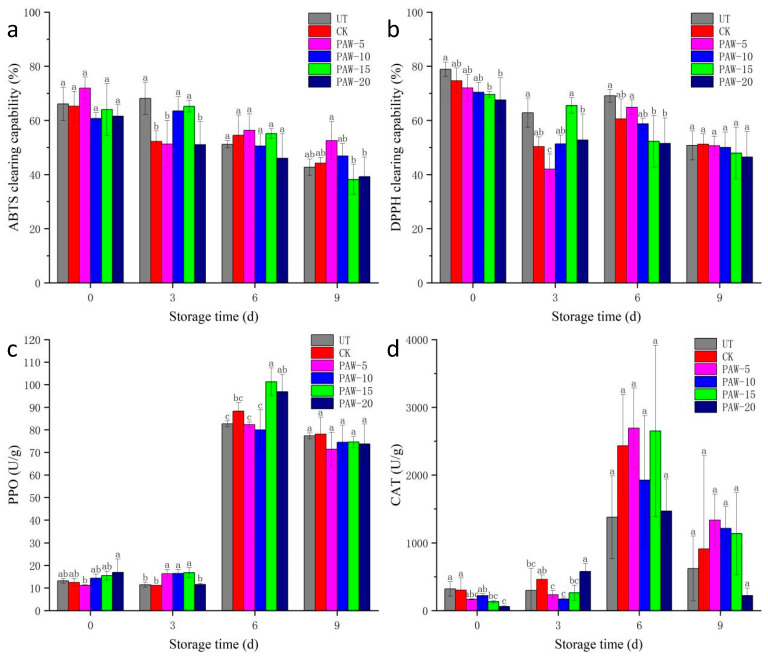
Effect of PAW on the antioxidant activity and enzyme activity of shepherd’s purse during storage: (**a**) ABTS clearing capability, (**b**) DPPH clearing capability, (**c**) PPO values, and (**d**) CAT values. a–c represents significant differences among different treatments at the same storage time (*p <* 0.05). UT (control); CK (control treatment immersed in 1 L of distilled water for 10 min); PAW-5 (control treatment immersed in 1 L of 5 min activated water for 10 min); PAW-10 (control treatment immersed in 1 L of 10 min activated water for 10 min); PAW-15 (control treatment immersed in 1 L of 15 min activated water for 10 min); PAW-20 (control treatment immersed in 1 L of 20 min activated water for 10 min); replicates (*n* = 3).

In order to analyze the antioxidant activities of the shepherd’s purse with the different PAW treatments, ABTS and DPPH clearing capabilities were determined. Figure 5a,b indicates the ABTS and DPPH clearing capabilities of the shepherd’s purse. For two assays, there were differences between the samples with the different PAW treatments. The trends of ABTS clearing capabilities of the PAW-10 and PAW-15 samples were the same as that of the UT sample, and the PAW-20 sample had the lowest ABTS clearing capability during the whole storage period. While a slight reduction in the ABTS clearing capability of the PAW-5 sample was found during early storage (0–3 days), the PAW-5 sample exhibited a higher ABTS clearing capability after 6 days of storage. Consistent with the change of the ABTS clearing capability, the DPPH clearing capability of the PAW-5 sample was reduced at early storage (0–3 days) and then increased (6–9 days). Compared with the UT and CK samples, the DPPH clearing capabilities of the PAW-15 and PAW-20 samples were lower after 6 days of storage; the PAW-10 sample had a higher DPPH clearing capability at the end of storage. The results were in accordance with a previous study [31]. The behavior of antioxidant activity was similar to the observed behavior of ascorbic acid, which may be the main factor determining antioxidant activity. Similar findings were reported by Rodriguez, Gomes, Rodrigues, & Fernandes [33].

The PPO and CAT activities of the shepherd’s purse during the storage are shown in Figure 5c,d. The PPO activity of samples with different treatments showed an increasing trend during storage. The CAT activity of samples with different treatments increased and then decreased, which reached the highest values at 6 days of storage. A similar finding was reported by Chen, Hu, Zhang, Jiang, & Liu [34]. As shown in Figure 4c, although the PPO activities of the PAW-15 and PAW-20 samples showed higher values than that of the UT and CK samples at 6 days of storage, the PPO activities of samples with PAW treatment remained lower than that of the UT and CK samples, with no obvious difference at the end of storage. As shown in Figure 4d, the UT and PAW-20 samples had lower CAT activities during the storage period, while the CAT activities of the PAW-5, PAW-10, and PAW-15 samples were higher than those of the UT and CK samples after 9 days of storage. In summary, PAW treatment had no obvious influence on the PPO activity and could increase the CAT activity of samples at 9 days of storage. This result was in agreement with the study by Xiao et al. [35], who suggested that PAW could inhibit the increase in PPO activity and decrease in CAT activity of samples during the later stage of storage.

### 3.5. Effect of PAW Treatment on the Microbial Growth of Shepherd’s Purse

**Figure 6:**
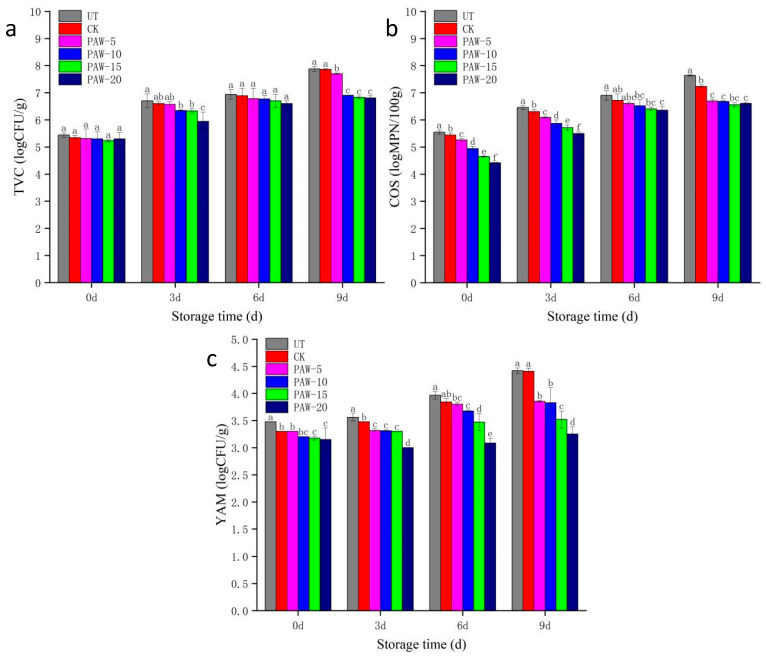
Microbial growth of shepherd’s purse during 9 days of storage: (**a**) TVC, (**b**) COS, and (**c**) YAM. a–f represents significant differences among different treatments at the same storage time (*p <* 0.05). UT (control); CK (control treatment immersed in 1 L of distilled water for 10 min); PAW-5 (control treatment immersed in 1 L of 5 min activated water for 10 min); PAW-10 (control treatment immersed in 1 L of 10 min activated water for 10 min); PAW-15 (control treatment immersed in 1 L of 15 min activated water for 10 min); PAW-20 (control treatment immersed in 1 L of 20 min activated water for 10 min); replicates (*n* = 3).

The effect of PAW on the counts of total bacteria, coliform, yeast, and mold of shepherd’s purse during storage periods is shown in Figure 6. Compared to the UT and CK samples, PAW treatment obviously reduced the counts of bacteria, coliforms, yeast, and mold during the whole storage period. There was no difference between the UT and CK samples on the counts of bacteria and coliform. The bactericidal efficiency was influenced by the time of PAW treatment; that is, an increase of PAW treated time from 5 min to 20 min resulted in a gradual decrease of microbial populations. Among all the tested microbial groups, coliform was the most sensible one, and it was reduced by 0.29 LogCFU/g, 0.61 LogCFU/g, 0.90 LogCFU/g, and 1.13 LogCFU/g after PAW treatment for 5, 10, 15, and 20 min at 0 days of storage compared with those in the UT sample, respectively. Therefore, it would be worth emphasizing in the text that a change in log by one unit results in a ten-fold change in the level of microorganisms. In addition, the microbial growth of samples treated with PAW for 5, 10, 15, and 20 min decreased during storage periods. Compared with the UT samples, treatment with PAW for 5–20 min reduced the number of bacteria, coliform, yeast, and mold by 0.18–0.94, 0.59–0.97, 0.90–1.18, and 1.03–1.17 LogCFU/g at 9 days of storage, respectively. Similar findings were obtained for fresh-cut lettuce and button mushrooms; PAW treatments achieved an effective reduction in bacterial and fungal populations during storage [36].

### 3.6. Effect of PAW Treatment on the Aroma Profiles of Shepherd’s Purse

**Figure 7:**
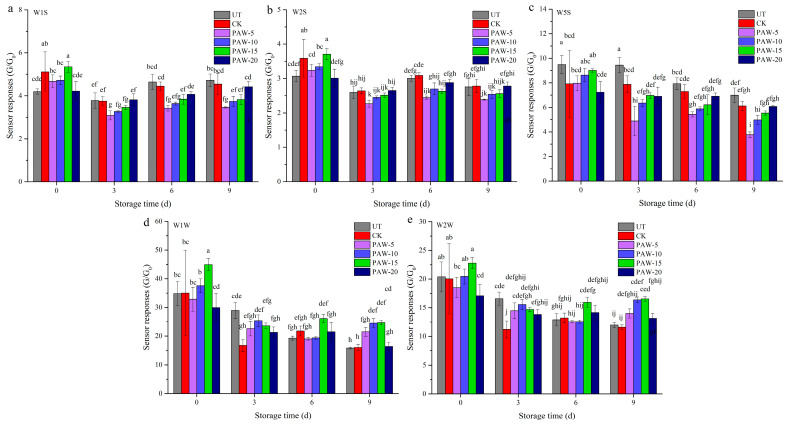
Effect of PAW treatment on aroma profiles of shepherd’s purse during 9 days of storage: (**a**) W1S, (**b**) W2S, (**c**) W5S, (**d**) W1W and (**e**) W2W. a–j represents significant differences among different treatments at the same storage time (*p <* 0.05). UT (control); CK (control treatment immersed in 1 L of distilled water for 10 min); PAW-5 (control treatment immersed in 1 L of 5 min activated water for 10 min); PAW-10 (control treatment immersed in 1 L of 10 min activated water for 10 min); PAW-15 (control treatment immersed in 1 L of 15 min activated water for 10 min); PAW-20 (control treatment immersed in 1 L of 20 min activated water for 10 min); replicates (*n* = 3).

An e-nose can be used to characterize and differentiate the aroma profiles of the shepherd’s purse with different treatments. The response values of e-nose sensors of different samples are presented in Figure 7. The W1C, W3C, W3S, W5C, and W6S sensors showed lower responses to the samples, and there was no marked difference in responses among different samples throughout storage (not shown). Meanwhile, W1S, W2S, W5S, W1W, and W2W sensors had larger and different responses of all samples during storage, indicating that the shepherd’s purse may have high concentrations of sulfides, terpenes, alcohols, nitrogen oxides, and aromatic compounds. The response values of W1S and W2S of all samples were reduced at early 3 days of storage and then increased after 6 days of storage. Sensor W5S of all samples showed a decreasing evolution of response during storage periods. The sensors W1W and W2W of the UT, CK, and PAW-20 samples showed decreasing trends of responses throughout storage periods, and PAW-5, PAW-10, and PAW-15 samples reduced at early 3 days of storage and then increased after 6 days of storage. In particular, PAW-5, PAW-10, and PAW 15 samples had lower response values of W1S, W2S, and W5S than the UT, CK, and PAW-20 samples during storage periods, while they showed higher response values of W1W and W2W after 9 days of storage. The results indicated that PAW treatments for 5, 10, and 15 min inhibited the formation of terpenes, alcohols, and nitrogen oxide compounds in samples and maintained higher concentrations of sulfur compounds in samples during the later stage of storage. Similar findings were reported by Cortellino, Gobbi, & Rizzolo [37]. The highest response values of W1S, W2S, and W5S were obtained in fresh-cut apples, and the values increased throughout the 10 days of storage. These sensors could be used to monitor the shelf-life of fruit products which are most sensitive to volatile compounds related to the microflora metabolism or aerobic activity [38].

### 3.7. Effect of PAW Treatment on the Microstructure of Shepherd’s Purse

**Figure 8:**
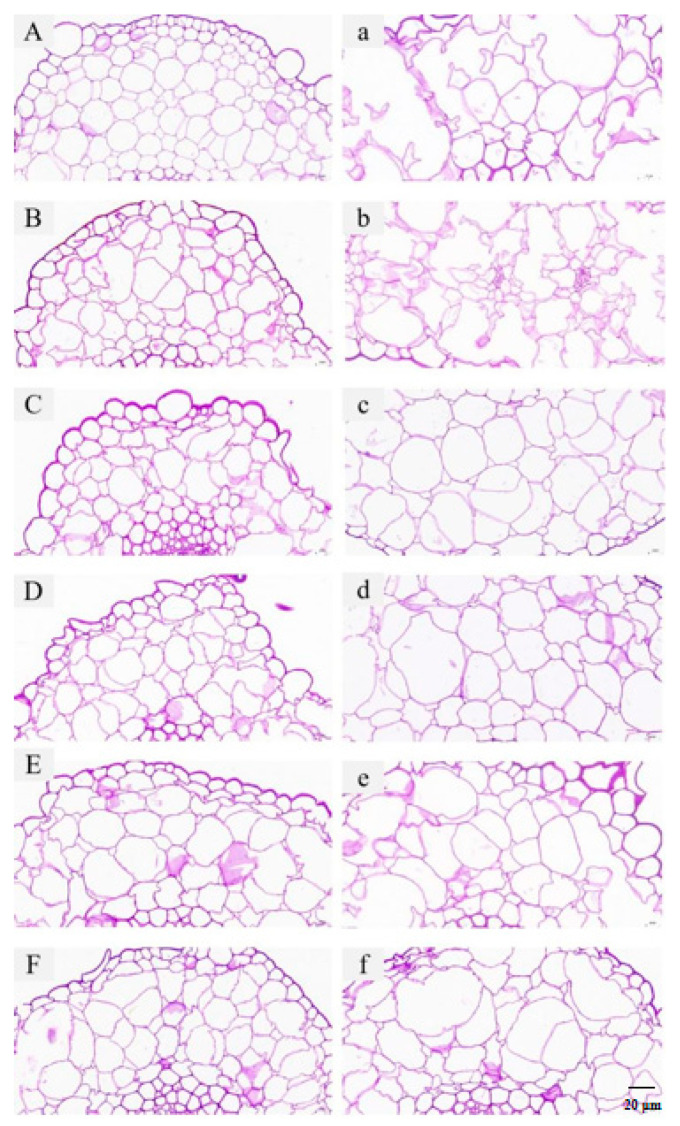
Microstructural images of UT (**A**), CK (**B**), PAW-5 (**C**), PAW-10 (**D**), PAW-15 (**E**), and PAW-20 (**F**) samples at 0 days of storage, and that of UT (**a**), CK (**b**), PAW-5 (**c**), PAW-10 (**d**), PAW-15 (**e**), and PAW-20 (**f**) samples at 9 days of storage, respectively. The magnification was set at 40×. UT (control); CK (control treatment immersed in 1 L of distilled water for 10 min); PAW-5 (control treatment immersed in 1 L of 5 min activated water for 10 min); PAW-10 (control treatment immersed in 1 L of 10 min activated water for 10 min); PAW-15 (control treatment immersed in 1 L of 15 min activated water for 10 min); PAW-20 (control treatment immersed in 1 L of 20 min activated water for 10 min).

Microstructural images of the shepherd’s purse with different treatments obtained by light microscope are shown in Figure 8. At 0 days of storage, dense, small, and regular-shaped cells were clearly visible in the image of the UT sample (Figure 8A), while cells from the CK sample became slightly swollen (Figure 8B). This might be attributed to the fact that samples immersed in distilled water gained water [39]. Moreover, there was no obvious difference in microstructure between the PAW-5, PAW-10, and CK samples (Figure 8B–D), and larger volume of cells in the PAW-15 and PAW-20 samples at 0 days of storage were obtained (Figure 8E,F). According to this phenomenon, PAW treatment influenced the microstructures of samples when a higher intensity of PAW was used with the same immersion time. This was similar to previous research on the PAW treatment of bananas [40]. After 9 days of storage, a breakdown of the cell wall outline and the cleavage of the intracellular network was observed in the UT sample (Figure 8a), and the decomposition of cell walls became more severe in the CK sample (Figure 8b). In contrast, the cell walls and intracellular networks were still visible in samples with PAW treatment, while the intercellular space and volume of the cells increased compared with samples at 0 days of storage. However, differences in microstructures were obtained for samples treated with different activation times of PAW. As shown in Figure 8c–f, the cell was relatively intact, the intercellular space was small in the PAW-5 sample, and the cell walls of samples gradually decomposed with an increasing PAW activation time. Therefore, a longer activation time of PAW may cause chemical damage to the surface microstructure of the cells due to the increase of reactive substances [41].

### 3.8. Correlation Analysis between PAW Treatments and the Postharvest Quality of Shepherd’s Purse

**Figure 9:**
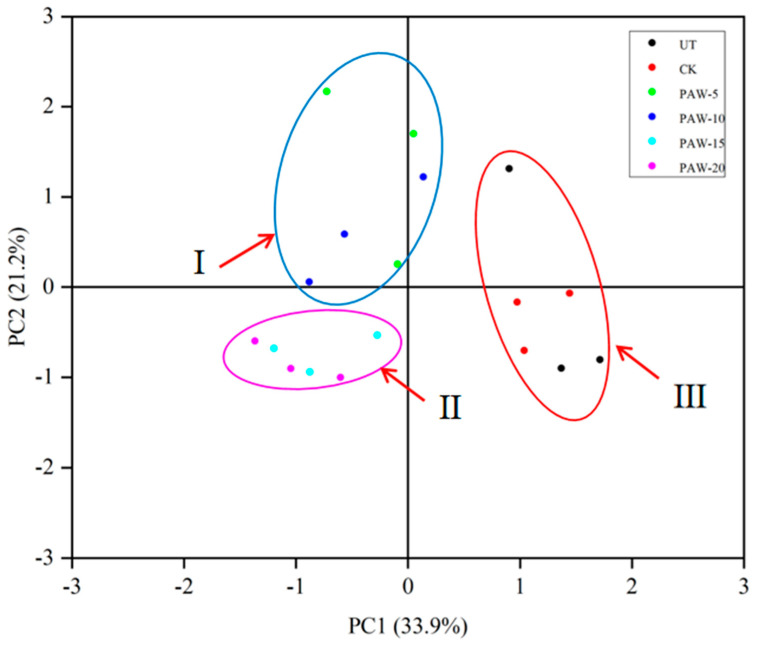
Correlation analysis between PAW treatments and the postharvest quality of shepherd’s purse on 9 days of storage. (I) PAW-5 and PAW-10 treatment, (II) PAW-15 and PAW-20 treatment, (III) UT and CK treatment, UT (control); CK (control treatment immersed in 1 L of distilled water for 10 min); PAW-5 (control treatment immersed in 1 L of 5 min activated water for 10 min); PAW-10 (control treatment immersed in 1 L of 10 min activated water for 10 min); PAW-15 (control treatment immersed in 1 L of 15 min activated water for 10 min); PAW-20 (control treatment immersed in 1 L of 20 min activated water for 10 min); replicates (*n* = 3).

To further investigate the impact of the PAW treatments on the postharvest quality of the shepherd’s purse, we conducted a PCA analysis of the obtained results on the ninth day of storage (Figure 9). The PCA plot reveals valuable insights, with PC-1 accounting for 33.9%, PC-2 for 21.2%, and the cumulative contribution rate reaching 56.1%. These values effectively capture the essential information from the original data. Figure 9 clearly demonstrates significant distinctions between the distribution of various samples within the PCA plot. Notably, the samples exhibit three distinct levels of PAW treatment. The PAW-5 and PAW-10 treatment methods closely resemble the parameter indicators of PAW-15 and PAW-20 in the second principal component, indicating their similarity in terms of relevant parameters. Conversely, the UT and CK processing methods are entirely separable from other treatment methods along the first principal component. It can be seen that the second axe (PC2) separates the low and high PW doses. Hence, by using principal component analysis to distinguish between the processing methods, the goal of distinguishing pouch samples under different processing methods can be achieved.

## 4. Conclusions

In conclusion, this study comprehensively expounded the effects of PAW on the postharvest quality of shepherd’s purse. The appropriate dosage of PAW inhibited the yellowing and reduced the weight loss of the shepherd’s purse, meanwhile improving its quality. Based on the results of the ascorbic acid, chlorophyll, TPC, TFC, ABTS, DPPH, PPO, and CAT, treatment with PAW-5 and PAW-10 reduced the nutritional loss of shepherd’s purse. Additionally, the PAW treatment maintained higher antioxidant activities and inhibited the decrease of antioxidant components of the samples during storage. Moreover, the PAW treatments obviously reduced the counts of bacteria, coliforms, yeast, and mold during storage periods. In general, PAW treatments effectively improved the quality of the shepherd’s purse after harvesting. The results indicated that PAW treatment might be a promising approach to help improve the microbiological safety and storage quality of shepherd’s purse.

## Figures and Tables

**Table 1:** Gas sensor array species.

Sensor No.	Sensing Species
W1C	Aromatic hydrocarbons, benzene hydrocarbon
W5S	Nitrogen oxides
W3C	ammoniac compounds, aromatic compounds
W6S	Mainly hydrogen
W5C	Short-chain alkanes, aromatic compounds
W1S	Methyl compound
W1W	Sulfur inorganic compounds
W2S	Alcohol, ether, aldehydes, ketones
W2W	Aromatic compounds, sulfur organic compounds
W3S	Long-chain alkane

## Data Availability

The original contributions presented in the study are included in the article, further inquiries can be directed to the corresponding author.
